# Cellular Mechanisms of Coronary Artery Spasm

**DOI:** 10.3390/biomedicines10102349

**Published:** 2022-09-21

**Authors:** Beata Franczyk, Jill Dybiec, Weronika Frąk, Julia Krzemińska, Joanna Kućmierz, Ewelina Młynarska, Magdalena Szlagor, Magdalena Wronka, Jacek Rysz

**Affiliations:** Department of Nephrology, Hypertension and Family Medicine, Medical University of Lodz, ul. Żeromskiego 113, 90-549 Łódź, Poland

**Keywords:** coronary artery spasm, cellular mechanism, endothelial dysfunction, oxidative stress, smooth muscle hypercontractility, inflammation, atherosclerosis, thrombosis, variant angina

## Abstract

Coronary artery spasm (CAS) is a reversible phenomenon caused by spontaneous excessive vascular smooth muscle contractility and vascular wall hypertonicity, which results in partial or complete closure of the lumen of normal or atherosclerotic coronary arteries. The clinical picture of CAS includes chest discomfort which is similar in quality to that of stable effort angina. Mechanisms underlying the development of CAS are still unclear. CAS certainly is a multifactorial disease. In this review, we paid attention to the role of the main pathophysiologic mechanisms in CAS: endothelial dysfunction, chronic inflammation, oxidative stress, smooth muscle hypercontractility, atherosclerosis and thrombosis, and mutations leading to deficient aldehyde dehydrogenase 2 (ALDH2) activity. These findings might shed novel insight on the underlying mechanisms and identify potential diagnostic and therapeutic targets for cardiovascular diseases in the future.

## 1. Introduction

Coronary artery spasm (CAS) is a reversible phenomenon caused by spontaneous excessive vascular smooth muscle contractility and vascular wall hypertonicity, which results in partial or complete closure of the lumen of normal or atherosclerotic coronary arteries [1,2]. The concept of CAS was first postulated by Prinzmetal et al. who described angina that occurs at rest or during regular daily activities which could not be explained by the increased oxygen demand of the myocardium [3]. Prevalence of CAS is diverse between countries: in the Japanese, 24.3%, followed by the Taiwanese, 19.3%, and Caucasian, 7.5%, populations [2]. Among patients aged 40 to 70 years, CAS is more common in men than in women [1]; however, it is mostly a disease of middle- and older-aged men and post-menopausal women [4].

The clinical picture of CAS includes chest discomfort which is similar in quality to that of stable effort angina [4]. A typical CAS attack is transient, often lasts only a few seconds, and is unpredictable; however, it arises particularly from midnight to early morning [4,5]. It occurs at rest and is a vague sensation of compression in the precordium or upper abdomen with radiation mostly to the neck, jaw, and left shoulder [6]. Angina may be accompanied by cold sweats, syncope, and a lowering of blood pressure [6]. Kishida et al. revealed that 82% (872 of 1062 episodes) of CAS episodes were asymptomatic and that syncope occurred in 12.5% (30 of 240 patients) of patients with CAS [7].

The main risk factors for CAS are smoking, age, high-sensitivity C-reactive protein (hs-CRP), hypertension, LDL cholesterol, and diabetes mellitus [1,2].

Mechanisms underlying the development of CAS are still unclear. CAS certainly is a multifactorial disease [5]. The main pathophysiologic mechanisms in CAS are dysfunction of the autonomic nervous system, endothelial dysfunction, chronic inflammation, oxidative stress, smooth muscle hypercontractility, atherosclerosis and thrombosis, and mutations leading to deficient aldehyde dehydrogenase 2 (ALDH2) activity [1].

In patients with CAS due to dysfunction of the endothelium, deficiency in nitric oxide (NO) is observed [8]. One of the damaging factors for the endothelium is oxidative stress. Free radicals degrade NO which results in artery spasms. Cigarette smoking is one of the main risk factors that intensify this process [9]. In addition, it is critical for people suffering from CAS to cease smoking, as the inflammation in the body, an essential part of smoking, triggers coronary spasms [10,11,12,13].

Fortunately, it has been reported that antioxidants such as vitamin C or E can restore disturbed arterial reactivity [14,15]. Furthermore, estrogen, a hormone responsible for enhancing NO synthase activity, can be considered as a protective factor. It has been shown that high estrogen levels (typical of the pre-menopausal period) were associated with a lower frequency of ischemic episodes [16].

One of the triggers of CAS is also vascular smooth muscle cell hyperreactivity. The excessive intracellular influx of calcium ions, disturbances in the functioning of calcium channels, and malfunctioning of ATP-sensitive potassium channels may result in the occurrence of coronary artery spasms [17,18,19]. RhoA/Rho-kinase (ROK) activity and a number of neurotransmitters are also involved in the pathogenesis of hypercontractility leading to CAS [2,20,21,22,23].

Another important risk factor is also deficiency of magnesium, an endogenous calcium channel antagonist [1,4,5,24]. Intravenous magnesium administration is beneficial in patients with CAS [5].

**Figure 1:**
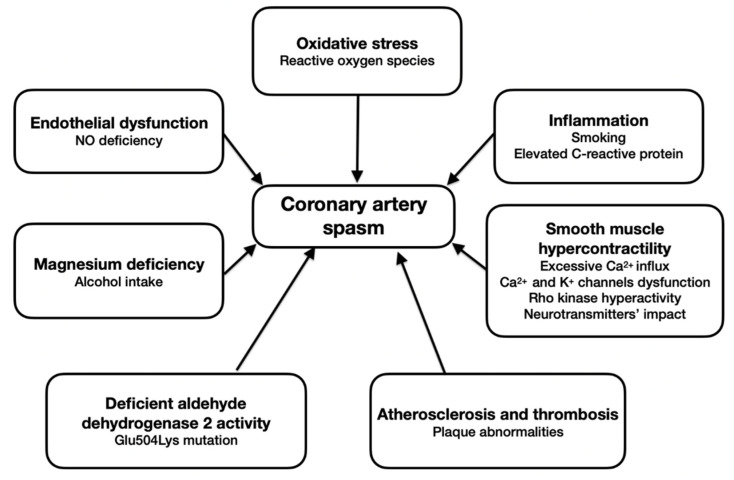
Pathogenetic mechanisms of CAS.

The main pathogenetic mechanisms of CAS are presented in Figure 1.

Lifestyle change and elimination of risk factors, as well as adherence to prescribed pharmacotherapy, form the basis of the management of CAS and reduce the risk of further episodes in the future [25]. The pharmacotherapy of CAS is based on the use of calcium channel blockers (CCBs) and/or nitrates. In exceptional cases, invasive therapies can be used [26].

Diagnosis of vasospastic angina may be problematic [5]. The primary role in the initial evaluation of a patient with an attack of a coronary artery spasm is to perform an electrocardiography (ECG) [5]. However, coronary angiography with a provocation test is considered the gold standard for diagnosing the disease. There are also other modern imaging tests such as intravascular ultrasound (IVUS) and optical coherence tomography (OCT), which are much more accurate but less commonly used [1]. 

## 2. Endothelial Dysfunction

The vascular endothelium is known as a regulatory organ that is essential for the proper function of the cardiovascular system. Due to its ability to produce biologically active substances, the endothelium is a significant factor that maintains homeostasis. Moreover, it is crucial for the fluidity of blood because of its anticoagulant, fibrinolytic, and antithrombotic properties. Therefore, its dysfunction plays an important role in the pathomechanism of blood vessel alterations [27].

One of the multiple roles of a normal functional endothelium is the production of NO. This compound is responsible for vasodilatation by suppressing vasoconstrictors such as angiotensin II and endothelium I [1]. Furthermore, NO deficiency may intensify their synthesis [28,29].

In young healthy people, acetylcholine (ACh) induces an increase in coronary artery diameter by releasing NO [8]. Nevertheless, there are other endothelium-dependent vasodilators such as serotonin, histamine, or ergonovine which are also a virtue of nitric oxide-releasing mechanisms [30]. On the other hand, a dysfunctional endothelium is characterized by the deficiency of NO. Consequently, in subjects with coronary atherosclerosis, intracoronary infusion of ACh results in spasms [8]. The difference between those two groups proved useful in the diagnosis of CAS. Injections of ACh are used as a provocative test [4]. However, it has been reported that coronary hyperconstriction induced by ACh involves all coronary segments. Spasms caused by ergonovine or serotonin concern only the given coronary site or segment which is similar to spontaneous spasms. Due to this fact, ACh may not be an appropriate option [10].

Nonetheless, nitrates via conversion into NO in vivo are independent of endothelial mechanisms. Synthesis of NO from L-arginine can be inhibited by L-monomethyl-arginine (L-NMMA) [30]. Kugiyama et al. [31] conducted a study in which they infused L-NMMA into coronary arteries in 21 patients with coronary spastic angina (CSA) and in 28 control patients. A coronary spasm was induced by ACh. Administration of L-NMMA in the control group resulted in a decrease in the basal diameter of a coronary artery but ended up with no effect in the other group. Moreover, the dilator response to nitroglycerin was significantly higher in patients with CSA. This is a result of the super-sensitivity of spasm arteries to nitroglycerin. It might be due to the deficiency of endogenous NO activity.

According to Kawano’s research [16], variation in estrogen levels is strictly connected with the frequency of myocardial ischemia. As it is known, estrogen is responsible for enhancing NO synthase activity [32]. Due to the similarity between the endothelial function of a brachial and coronary artery [33], flow-mediated dilation of the brachial artery was assessed in the study. This magnitude is mostly based on endothelium-derived nitric oxide [34], and as Kawano showed, it is related to the variation in estradiol levels during the menstrual cycle. The ischemic episodes occurred more frequently with low estrogen levels and less frequently at high estrogen levels. This is why CAS occurs more often in post-menopausal women. However, no similar association was demonstrated in progesterone levels. 

## 3. Oxidative Stress

Oxidative stress is the state of imbalance between the action of reactive oxygen species (ROS) and the biological ability to dispose of reactive intermediates or to repair the damage. Due to enzyme activity, the reducing environment in cells is maintained. Disruption of this mechanism can contribute to the production of free radicals and peroxides. Free radicals are defined as substances that have one or more unpaired electrons. This feature makes them highly reactive and allows them to donate their electrons to other molecules. Consequently, it leads to chain reactions and then oxidative damage [35].

Studies showed that oxidative stress plays an important role in the pathogenesis of endothelial dysfunction [36,37]. ROS are responsible for the degradation of NO, thus stimulating vasoconstriction and causing endothelial damage [30].

Thioredoxin is a ubiquitous enzyme, and one of its functions is cytoprotection against oxidative stress [38]. Miyamoto et al. [39] reported that plasma levels of thioredoxin were increased in subjects with coronary spastic angina. Furthermore, it was shown that higher thioredoxin levels were strictly connected with a more frequent occurrence of anginal attacks. It can be concluded that the high activity of the disease is associated with intensified oxidative stress.

Smoking has been recognized as one of the major risk factors for coronary spasms [9]. Cigarette smoke is the source of a large number of free radicals causing the degradation of NO [40]. It has been reported that the number of smokers was significantly higher in the coronary spastic angina group than in the chest pain syndrome group [39]. Oxidative activity of estrogen protects pre-menopausal women from CAS, but this does not apply to those who smoke [16]. Motoyama et al. [14] concluded that vitamin C can improve impaired endothelium-dependent vasodilation in chronic smokers. Serum levels of vitamin C were lower in smokers than in nonsmokers. Additionally, plasma levels of thiobarbituric-acid-reactive substances (TBARS) were remarkably higher in those addicted to cigarettes. TBARS are known as an indicator of oxidative stress. However, the infusion of vitamin C resulted in a decrease in TBARS levels in smokers but did not change the levels in nonsmokers. These results are presented in Table 1.

Not only vitamin C is helpful for patients with coronary spastic angina. Studies have also shown that vitamin E is able to restore disturbed arterial reactivity. Miwa et al. [41] concluded that plasma vitamin E levels were markedly lower in subjects suffering from active variant angina than in those without coronary spasms. This finding suggests a connection between oxidative stress and CAS. In Motoyama’s research [15], the effect of the oral administration of 300 mg/day of vitamin E on endothelium-dependent vasodilation was examined. It was shown that supplementation of vitamin E resulted in improvement in flow-dependent vasodilation. Furthermore, this management also caused a decrease in plasma TBARS levels.

The mechanism of either vitamin C and E is based on increasing the availability of intracellular reduced glutathione (GSH) and thiols [42]. Glutathione is mainly responsible for protection from oxidative stress and the prevention of nitric oxide inactivation. It has been reported that intracoronary infusion of GSH can restore the proper function of the endothelium [43] and suppress constrictor response to ACh in epicardial coronary arteries [44].

## 4. Inflammation 

The evidence based on many studies [4,10,11,45], as well as clinical settings, suggests an association between CAS and inflammation, especially connected with Rho-kinase regulation [10,11]. CAS indicates an association with chronic inflammation by elevated biomarkers such as hs-CRP [2,12,45], interleukin-6, peripheral leukocytes, monocytes [2], and soluble CD40 ligands [12]. Moreover, adhesive molecules, such as P-selection, are elevated in patients with CAS [46]. A chronic low-grade inflammatory state may lead to CAS through RhoA/Rho-kinase pathway activation and a reduction in endothelial NO activity [11]. C-reactive protein, a sensitive marker of inflammation, suppresses endothelial NO activity and activates RhoA signaling [47] and Rho-kinase activity in white blood cells, which is a prognostic factor for the severity of CAS, correlating with the Il-6 level in the plasma [2]. Moreover, another inflammatory marker, an increased mastocyte level, has been reported in patients with CAS [48]. Cigarette smoking, a major risk factor for coronary artery spasms, is connected with low-grade inflammation and an increased hs-CRP level [12,13]. Thus, it confirms that even minor elevations of the hs-CRP level in serum are essentially and independently associated with coronary spasms. Furthermore, a recent study suggested that coronary spasms are associated with inflammation of coronary adventitia and perivascular adipose tissue [49].

## 5. Smooth Muscle Hypercontractility

The activity of vascular smooth muscles, contraction and relaxation, is regulated by the phosphorylation and dephosphorylation of the myosin light chain (MLC). Physiologically, phosphorylation is induced by an increase in the intracellular concentration of calcium ions, which, being in a complex with calmodulin, activate myosin light chain kinase leading to phosphorylation of MLC. In coronary artery spasms, excessive contraction of the smooth muscles of the coronary vessels occurs in response to an increase in the intracellular Ca^2+^ influx [4].

Elevated expression of L-type Ca^2+^ channels and an increase in Ca^2+^ entry into vascular smooth muscle cells (VSMCs) through the channels may also initiate the spasm [50]. Moreover, a Ca^2+^ influx through the alpha1H Ca^2+^ system is crucial to coronary arteries’ relaxation. The deficiency of α1HT-type calcium channels inhibits the relaxing effect of ACh [17], which may contribute to the pathogenesis of coronary artery spasms.

Phospholipase C overactivity, which is dependent on Ca^2+^, may also cause CAS through enhanced contraction of VSMCs [51].

ROK and RhoA, being VSMC contractility regulators, are involved in the pathogenesis of coronary artery spasms. Properly, the Rho-kinase metabolic pathway modulates the level of MLC phosphorylation by the inhibition of myosin phosphatase.

The hyper-reactivity of Rho kinase in smooth muscle cells promotes its contraction by sensitizing the myosin light chain to calcium ions, as well as indirectly increasing the phosphorylation of this chain, promoting vasoconstriction. A study on animal models showed that hydroxyfasudil, the Rho kinase inhibitor, prevented dose-dependent excessive coronary contractions, supporting the role of Rho kinase in the pathogenesis of CAS [52,53].

It is essential to mention that inflammation may be also a trigger for vascular smooth muscle cell hyperactivity. The activity of Rho kinase in the coronary artery can be increased by the proinflammatory mediator, interleukin 1β (Il-1β) [10], which confirms the participation of the inflammatory process in the generation of excessive smooth muscle contractions in CAS.

Coronary artery spasms may also be the result of a defect in the endothelial enzyme responsible for the production of NO, which is one of the key mediators inducing vasodilation. Rho kinase can inhibit the production of NO [54], and the absence of this relaxing factor might result in CAS. Treatment that shows the appropriate effect in this situation is statins, which increase the activity of endothelial NO, decrease ROS, and suppress the RhoA/ROCK pathway [2].

ATP-sensitive potassium channels (KATP) are responsible for the regulation of vascular tone. KATP channels are made up of two types of subunits: the lumen-forming subunit (usually Kir 6.2) and the sulfonylurea receptor (SUR). Studies [18,19] suggest that the SUR2 KATP channel is a crucial regulator of episodic vasomotor activity, and the loss of function of SUR2 KATP channels is of key importance for the proper function of the coronary vessels. The loss of KATP activity in the smooth muscles of the coronary vessels correlates with the occurrence of excessive contraction of the coronary artery. Based on these conclusions, it can be confirmed that there is a relationship between malfunctioning KATP channels and the occurrence of Prinzmetal variant angina.

Transmitters such as serotonin [20], dopamine [21], or histamine [22] may also be important factors influencing the VSMCs involved in the pathogenesis of CAS. Direct injection of ACh, a parasympathetic neurotransmitter of the nervous system, into the coronary artery also causes excessive contraction [23]. Focal administration of Il-1β may damage the inner membrane of the coronary vessels, sensitizing these places to the coronary administration of serotonin and histamine, which may lead to vasoconstriction [55].

There are multiple different pathways in which vascular smooth muscle hypercontractility may cause coronary artery spasms, but their connotation remains to be elucidated.

## 6. Atherosclerosis and Thrombosis

Atherosclerosis is characterized by the chronic accumulation of cholesterol-rich plaque in the arteries and is linked to a broad spectrum of cardiovascular diseases [56]. The disturbance of vascular endothelial structure and its function has also a major role in the pathogenesis of atherosclerosis [57,58].

In recent years, there has been substantial research showing correlations between atherosclerosis and vasomotor dysfunction. Although normal vessels are not excluded, CAS occurs more frequently among arteries with atherosclerotic segments [59]. Pelligrini et al. found that patients with CAS have more advanced atherosclerosis and a higher prevalence of vulnerable plaques, compared to those without CAS [60]. Furthermore, many studies have revealed that the presence of atherosclerosis together with CAS is associated with worse patient outcomes [59]. It is also worth mentioning that the CAS could trigger the rapture of stable plaque. In this way, coronary thrombosis and myocardial infarction (MI) might occur [61]. These findings by Yamagishi et al. suggest that atherosclerosis exists at the location of the focal vasospasm even in the absence of coronary disease on the angiography. Therefore, the development of focal vasospasms is linked to the presence of atherosclerotic lesions [62].

Spasms and atherosclerosis most likely have similar etiological pathways, such as endothelial dysfunction and arterial remodeling [63]. However, in accordance with recent research by Morita et al., atherosclerosis and CAS pathophysiologies might differ. Cigarette smoking, low diastolic blood pressure, and systemic low-grade inflammation are risk factors for CAS. On the contrary, the predictors for coronary atherosclerotic stenosis are age, diabetes mellitus, low HDL cholesterol, hypertension or high systolic blood pressure, and uric acid [64]. Interestingly, in the arterial vasculature, the atherosclerotic lesions develop at different locations where the spasm occurs. Moreover, recent studies showed that percutaneous coronary intervention (PCI) with stenting due to atherosclerotic stenosis does not contribute to the recurrence of CAS and that spasms often occur diffusely in the distal segments of the stented lesion [4].

CAS and atherosclerosis play an essential role in the pathogenesis of coronary heart diseases [65].

We also do not forget that coronary thrombosis is recognized as one of the causes of acute coronary syndromes including acute myocardial infarction, unstable angina, and sudden ischemic death [66,67]. Intimal tear, intimal erosion, and microthrombi are the three main abnormal morphologic findings of optical coherence tomography in patients with vasospasm-induced acute coronary syndrome as compared to individuals with chronic stable variant angina [68]. Kitano et al. established that spastic segments with focal spasms were more prone to have intracoronary thrombi [69]. Furthermore, intracoronary thrombi were found at all spastic segments, despite the presence of atherosclerotic lesions. Teragawa et al. indicated that CAS plays a significant role in thrombogenicity [70]. However, there were no significant differences between the spastic section with the intracoronary thrombi and the spastic segment in terms of the rate and severity of plaque formation as determined by CAS [70].

Patients with CAS have an increased risk of rapid plaque progression and ischemic events because coronary artery spasms trigger local thrombus formation and extensive inflammatory response [71]. Interestingly, patients with coronary spasms have increased plasma levels of hs-CRP and P-selection. Platelets are activated after attacks of CAS. Further, thrombogenesis is intensified [5]. Therefore, we can conclude that CAS might induce thrombosis in the coronary circulation.

## 7. Deficient Aldehyde Dehydrogenase 2 Activity

Susceptibility to disease is often genetically determined and is specific to a population [72]. This is also the case with CSA, which is a common disease among East Asians [73]. A mutation in the ALDH2 gene is believed to be the cause of this condition; moreover, it is likely that nearly 1 billion people worldwide carry the mutation, most of whom are Easterners [74]. It has been suggested that the risk of coronary heart disease and myocardial infarction may be increased due to the Glu504Lys variant in the ALDH2 gene, which is responsible for reducing the ability of ALDH2 to metabolize acetaldehyde [72]. East Asians are at higher risk for this variant, which is quite common in this population—it occurs in up to 40% of East Asians, while it is not observed in other parts of the world [73]. The mutation in question is a point mutation involving the substitution of glutamic acid for lysine and occurs on chromosome 12q24.2, leading to a mutant, dominant allele (A) [75]. Homozygotes carrying the defective variant are most at risk for enzyme deficiency, while heterozygotes have moderate enzyme deficiency [73]. It is also suggested that carriers of the A allele have a 48% risk of coronary artery disease (CAD) compared to those without the mutation [75].

Alcohol consumption has been proven to be one of the risk factors for the development of coronary heart disease [72]. After consuming ethanol, we can divide its metabolism into two stages. The first involves the conversion of alcohol to acetaldehyde with the involvement of alcohol dehydrogenases ADH; then, in the second stage, this product is oxidized to acetic acid via aldehyde dehydrogenases [72,74,75]. It is believed that acetaldehyde has 30 times more toxicity than ethanol; moreover, it forms free radicals that can react with DNA [74]. It is aldehyde dehydrogenase 2 that is thought to be the main enzyme that oxidizes the harmful aldehyde [72,76]. However, the action of ALDH2 is not limited to removing toxic acetaldehyde from the body but also aldehydes formed by lipid peroxidation [73]. Unfortunately, ALDH2 activity can be reduced by the presence of the 504lys variant, which leads to an increase in harmful acetaldehyde in the blood after ethanol ingestion due to impaired metabolism. As a result, homozygotes have an 18-fold higher concentration of noxious aldehyde, while heterozygotes have a 5-fold higher concentration of noxious aldehyde compared to wild-type homozygotes. The risk of CAD is likely related to the amount of alcohol consumed, with large amounts of alcohol having a higher risk [75]. It has also been suggested that high levels of acetaldehyde may affect circulation and blood pressure, thereby increasing the risk of CAD [72]. What is more, patients with ALDH2 deficiency are at risk for many other diseases such as esophageal and gastric cancer, cirrhosis, Alzheimer’s disease, and osteoporosis [74].

Mizuno Y et al. [73] investigated the relationship between smoking and the presence of a defective ALDH2 variant in the pathogenesis of CSA. The study showed that Asians with defective ALDH2*2 alleles have a higher risk of CSA. It was also noted that the genetic factor interacts with and exacerbates the deleterious effects of smoking on vasoconstriction, and the joint effect of the two factors interacts more strongly than each factor alone by increasing reactive aldehydes. It has been pointed out that reactive aldehydes may be a target for prevention and treatment in people at risk or suffering from CSA [73]. On the other hand, Li Y et al. [76] conducted a meta-analysis in which they evaluated the association between the G487A polymorphism of the ALDH2 gene and CAD in the Chinese population. They found a positive correlation of this gene variant with susceptibility to CAD [76]. Gu J. et al. [72] also conducted a meta-analysis in which they examined the relationship between the ALDH2 Glu504Lys polymorphism and the risk of CAD or myocardial infarction among the Asian population. Interestingly, it was shown that the Chinese and Korean population with the 504lys variant has a higher risk of developing CAD and MI which was not observed in the Japanese population [72]. Zhang L. et al. [75] evaluated the relationship between ALDH2 polymorphism and the risk of CAD. They analyzed 11 population-based studies, which included Chinese, Korean, and Japanese. Based on the study, they suggested that a defective dominant A allele is associated with lower concentrations of high-density lipoprotein C, which may also influence high CAD risk [75]. Another topic of interest was addressed by Fujioka K. et al. [74] who evaluated whether administration of the dietary supplement ESSENTIAL AD2 affects acetaldehyde levels in ALDH2-deficient subjects after alcohol consumption. They studied 12 subjects who were heterozygotes for mutations in the ALDH2 gene. Interestingly, after 28 days of supplement therapy, a reduction in serum acetaldehyde levels was observed after alcohol consumption. A reduction in liver enzymes was also observed during the study [74]. The Table 2 analyzes some of the studies discussed [72,73,74,75,76].

## 8. The Role of Magnesium

Magnesium levels in the body may be related to the occurrence of CAS. Its deficiency is one of the factors causing coronary vasospasms [1,4,5,24]. Magnesium, as an endogenous calcium antagonist [4,5], causes blockage of calcium channels, and therefore coronary smooth muscle contraction does not occur [24,26]. Compulsive alcohol consumption can also lead to angina attacks in a magnesium-dependent mechanism. This is due to magnesium deficiency caused by excessive urinary excretion of magnesium [24].

The basis of CAS is an increase in intracellular calcium ion levels and increased sensitivity to it. The increased sensitivity to calcium ions is due to the increased activity of the RhoA/ROCK pathway. It is also influenced by a decrease in NO release. As a result, coronary smooth muscle contraction occurs [5,24].

Magnesium supplementation is crucial in patients with low magnesium levels [1,4,5]. Beneficial effects of magnesium supply in patients suffering from CAS were reported to consist of a reduction in coronary vasospasms and spasm attacks and alleviation of hyperventilation-induced angina attacks [5,24,26].

An analysis of a case report by Popow et al. [77] describing coronary vasospasms caused by hypocalcemia (0.69 mmol/L; norm: 1.13–1.29 mmol/L) and hypomagnesemia (0.52 mmol/L; norm: 0.7–1.0 mmol/L) led to similar conclusions. The severe pain accompanying the pathology subsided after the infusion of calcium and magnesium. Although this is a rare cause of CAS, it should be taken into account during the differential diagnosis [77].

## 9. Diagnosis of Vasospastic Angina

The diagnosis of vasospastic angina (VSA) can be problematic [5]. Taking a clinical history and performing an ECG are the first step in the initial evaluation of a patient with an acute attack [78]. Patient symptoms are uncharacteristic and include transient retrosternal pain lasting a few seconds, occurring most often at rest, usually from midnight to the early morning hours [5]. ECG monitoring is important during an attack in the outpatient setting; however, it does not provide evidence of coronary artery spasms, and an attack may not occur at this time [5,79]. Changes in the ECG most often include the appearance of a peak T-wave; less often, we may see a lowering or elevation of the ST segment, as well as the appearance of a negative T-wave or U-wave [26,79]. These abnormalities are observed when the main coronary artery contracts partially or completely, while a mild spasm may not cause any changes in examination [26]. ECG-based diagnosis is often problematic because in many cases, several minutes after the episode occurs, parameters normalize [80]. In addition to the aforementioned changes during systole, we can observe various types of arrhythmias including supraventricular tachyarrhythmias, atrioventricular blocks, or ventricular tachycardias [26]. It is noteworthy that ventricular arrhythmias occur more frequently in patients with a shorter-lasting episode of ischemia, characterized by low severity [80].

**Figure 2:**
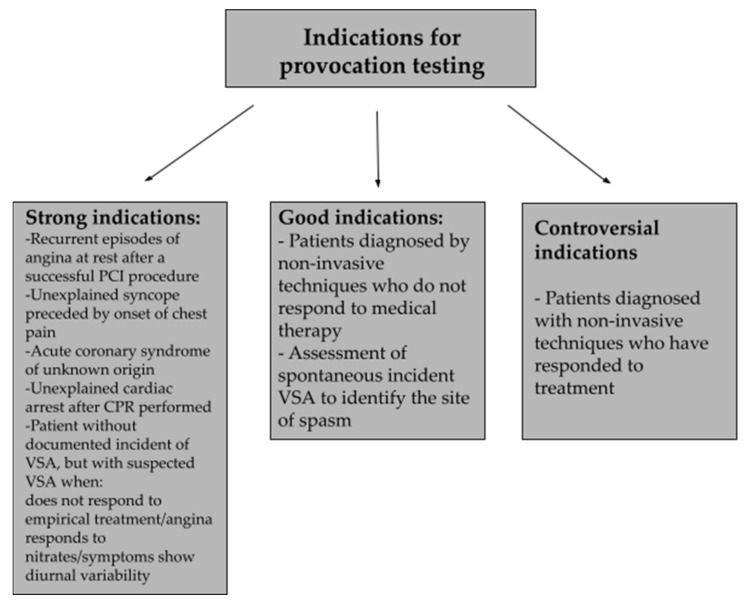
Some of the indications for the provocative test including their division [81].

Coronary angiography with provocation testing is considered the most convincing and reliable test for diagnosing VSA [1]. However, provocation testing is justified when we suspect VSA in a patient, but our suspicions have not been definitively confirmed [78]. The provocation test involves an intracoronary injection of vasoconstricting agents, among which ergonovine and acetylcholine are the most commonly used [1,26]. The test assesses the percentage of vessel lumen reduction, which can be 50%, 70%, 75%, or 90% [1,79]. Provocative tests have the ability to induce contraction in a given coronary vessel over a well-defined period of time, providing the opportunity to document and monitor this phenomenon in a laboratory setting [5]. Indications for a provocative test can be divided into strong, good, and controversial, and Figure 2 presents some of them [81].

Unfortunately, there are also several contraindications to the provocation test, some of which are listed in Table 3 [1].

**Figure 3:**
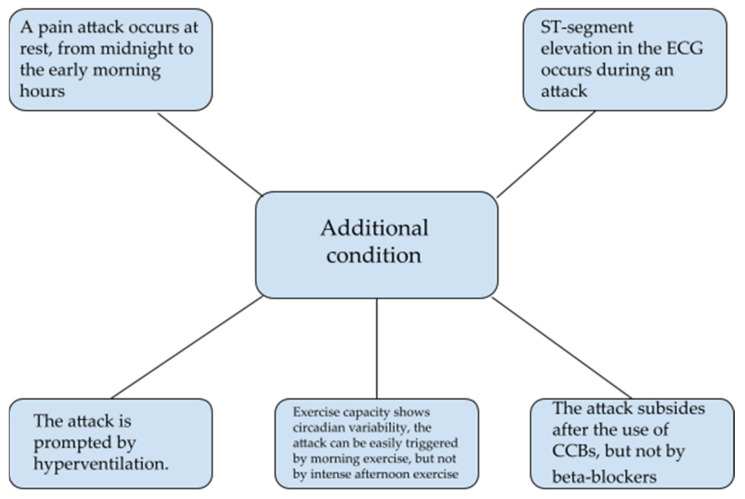
Additional criteria necessary for the diagnosis of angina pectoris when symptoms are relieved by nitroglycerin. Meeting one of the listed criteria is sufficient [5]. ECG, electrocardiography; CCBs, calcium channel blockers.

The diagnosis of the disease can also be made when fast-acting nitrate administered sublingually results in the resolution of ECG changes [5,79]. It is also possible when nitroglycerin causes a rapid relief of symptoms of the disease; however, in addition, at least one condition shown in Figure 3 must be met [5].

The use of cardiac biomarkers released during coronary artery contraction, such as creatinine kinase or troponins I and C, can be helpful in the diagnosis, but their levels are not always elevated [79]. If there are indications, a non-invasive exercise test can be performed, but only less than 30% of patients will have ischemic changes in the ECG resulting from exercise-induced vasospasms, and the rest of the patients will have a normal result [78]. Modern coronary artery imaging methods, such as IVUS and OCT, also appear to be useful in detailed diagnosis. IVUS visualizes the vessel’s thickened intima-media and even small atherosclerotic plaques at the site of the focal vasospasm; moreover, it detects lesions that may not be visible on angiography. OCT, on the other hand, allows imaging of structural changes such as erosions at the site of the coronary artery spasm [1].

Particularly noteworthy are the international diagnostic criteria presented by the International Study Group for Vasomotor Disorders in Coronary Artery Disease (COVADIS), which are helpful in the diagnosis of VSA. According to this group, diagnosis should be based on three basic pillars, among which are: (I) typical clinical signs, such as a spontaneous incident of nitrate-responsive angina, (II) ECG-documented myocardial ischemia during the spontaneous incident in the form of ST-segment elevation/decrease or new negative U-waves in at least two adjacent leads, and (III) documentation of coronary artery spasms [81].

## 10. Management of Coronary Artery Spasm

The basis of management of CAS is lifestyle changes and the elimination of risk factors [1,5,26]. It is recommended to stop smoking, consuming alcohol [1,4,5,26], and using substances such as cocaine [1,26]. Moreover, it is important to avoid emotional stress [1,4,5,26] and exercise in the early morning [1,4]. 

Another component of conservative treatment is pharmacotherapy. In the case of an acute CAS attack, nitrate is used sublingually or orally in spray form. Nitroglycerin or isosorbide dinitrate (ISDN) is converted to NO in vivo. Usage of nitrates is explained by the high sensitivity of the contracted coronary arteries to nitrates and the deficiency of endogenous NO [4,5]. Since these are short-acting drugs, it is necessary to use further preparations, which are CCBs [4]. CCBs are first-line treatment [1,26]. Both dihydropyridine and non-dihydropyridine CCBs have been shown to be effective in reducing recurrent angina [26]. However, some studies report that the use of non-dihydropyridine CCBs results in an almost complete reduction in CAS recurrence [1]. It is important that CCBs should be taken before sleep [4,5,82]. In a more selective (dihydropyridine) or less selective (non-dihydropyridine) way, CCBs act on the L-type calcium channels of myocytes in the vessels by inhibiting calcium influx. Thus, they reduce vascular resistance and cause coronary artery relaxation [83]. Long-acting nitrates are other drugs used to reduce the risk of angina [26], whereas the superiority of one drug over the other has not been demonstrated [1]. Since treatment with long-acting nitrates is accompanied by the phenomenon of tolerance, it is recommended to dose the drug in such a way that an 8-hour break is maintained [4,5,83]. Long-acting nitrates can be used both as monotherapy and as an adjunct to treatment with CCBs [26]. Underlying the action mechanism of nitrates is mitochondrial denitrification, which occurs in the vessel wall with the involvement of aldehyde dehydrogenase. As a result of these transformations, NO is produced. Hence, vasodilation occurs. The effect of nitrates varies with the dose used. Small doses cause a reduction in venous return and preload. In contrast, high-dose nitrates, similarly to CCBs, result in a decrease in afterload and thus a decrease in the heart’s oxygen demand while improving oxygen supply to the myocardium [83].

Statins are a group of drugs that cause CAS reduction and improve the prognosis of patients. The effect of statins is possible due to their properties that cause inhibition of the RhoA/ROCK pathway and an increase in NO activity [4]. They are an important adjunct to CAS pharmacotherapy [26]. Inhibitors of the RhoA/ROCK pathway, such as fasudil, a Rho-kinase inhibitor, may prove beneficial in the treatment of CAS because of the contraction-reducing properties of vascular smooth muscle cells (VSMCs). However, further studies are needed [4,5,26].

The use of aspirin in high doses, i.e., >325 mg per day, inhibits prostacyclin formation. This results in vasoconstriction; therefore, such treatment is contraindicated in CAS patients. In contrast, the use of low-dose aspirin, i.e., <100 mg per day, blocks the production of thromboxane A2, resulting in vasodilation; however, there are no conclusive reports on this topic. The use of low-dose aspirin in CAS patients is still a matter of debate [1,26].

Another drug with positive effects in CAS patients is Nicorandil. This drug causes coronary artery dilatation as a result of its nitrate- and potassium-channel-activating properties [1,4,5,83]. Nicorandil is recommended for patients with refractory CAS [26]. The use of magnesium in CAS patients and its mode of action has been discussed above, while antioxidants or estrogens have beneficial effects by improving endothelial function and reducing nitrate tolerance [4].

The use of alpha-1 adrenergic receptor antagonists is controversial, and it is thought that they may be a component of treatment when dealing with CAS refractory to conventional treatment [26]. Patients with CAS may benefit by taking magnesium or antioxidants (vitamins C and E) [1,4,5,26]. Meanwhile, in post-menopausal women, it is recommended to take estrogen, especially for patients with refractory CAS [4,5].

Drugs contraindicated in CAS include beta-blockers [1,26,82] but also, among others, catecholamines, parasympathetic stimulants, and ergot alkaloids [5,26]. They have vasoconstrictive effects and cause coronary vasospasms [1,5,82]. An exception is nebivolol, which owes its selectivity to β1 receptors and its ability to produce NO [1].

A summary of pharmacotherapy is shown in Table 4 [1,4,5,26,83].

Besides the non-invasive CAS treatment methods outlined, there are also invasive methods. Patients with atherosclerotic lesions may benefit from PCI and coronary artery bypass graft (CABG) [1]. Another method is implantation of a cardioverter-defibrillator (ICD). It is designed to prevent ventricular arrhythmias that can result in sudden cardiac death. However, it is suggested that this method of treatment should only be used in selected patients [26].

A study by Lin et al. [84] shows another potential treatment option for refractory CAS—sympathectomy. The study showed that sympathectomy, compared to traditional CAS treatment, was more effective in protecting against a syndrome of episodes of a major adverse cardiac event and death. However, the authors emphasize that further studies are needed [84].

## 11. Conclusions

Coronary arteries can contract and relax through several mechanisms. Hence, coronary constriction is not always pathological. Nevertheless, in some diseases, coronary constriction becomes more predominant and results in symptoms of a wide variety of heart and vasculature diseases.

In this review, we focused on the important molecular aspects of CAS. We paid attention to the role of endothelial dysfunction and oxidative stress, their pathomechanism, and their influence on the development of cardiovascular diseases. We also drew attention to the influence of smooth muscle hypercontractility, as an excessive intracellular influx of calcium ions, disturbances in the functioning of calcium channels, and malfunctioning of ATP-sensitive potassium channels may result in the occurrence of CAS. Moreover, the most recent discoveries have proven that inflammation plays a critical role in modulating all stages of CAS. The important role of atherosclerosis and thrombosis was also highlighted. Deficiency of aldehyde dehydrogenase 2 activity and magnesium contributes to CAS and was also considered to be of interest.

These findings might shed novel insight on the underlying mechanisms and identify potential diagnostic and therapeutic targets for cardiovascular diseases in the future.

## Figures and Tables

**Table 1:** Association between particular indicators and risk of CSA.

References	Study Design	Year	All Patients	Examined Indicator	Conclusions
Motoyama et al. [14]	Clinical trial	1997	40	vitamin C	Decreased vitamin C levels in smokers.
Motoyama et al. [14]	Clinical trial	1997	40	TBARS	Increased TBARS levels in smokers.
Miyamoto et al. [39]	Clinical trial	2004	170	thioredoxin	Increased thioredoxin levels in subjects with CSA.
Miwa et al. [41]	Clinical trial	1996	103	vitamin E	Decreased vitamin E levels in subjects with CSA.

TBARS, thiobarbituric-acid-reactive substances; CSA, coronary spastic angina.

**Table 2:** Association between mutation in the ALDH2 gene and CAD risk.

Authors	Gu et al. [72]	Mizuno et al. [73]	Fujioka et al. [74]	Zhang et al. [75]	Li et al. [76]
**Year**	2013	2017	2019	2015	2018
**Study design**	Meta-analysis	Clinical trial	Clinical trial	Meta-analysis	Meta-analysis
**All patients**	6762	410	12	8366	5644
**Aim of the study**	Evaluation of the association between the Glu504Lys mutation in the ALDH2 gene and the risk of CAD and myocardial infarction.	Evaluation of the correlation between smoking and the presence of ALDH2 gene mutation and their impact on CAD risk.	Evaluation of the effect of Essential AD2 supplementation by ALDH2-deficient subjects on blood acetaldehyde levels.	Evaluation of the correlation between polymorphisms of ALDH2 and CAD.	The effect of the G487A ALDH2 mutation on the occurrence of CAD.
**Conclusions**	The Glu504Lys mutation in the ALDH2 gene in the Asian population is associated with a high risk of myocardial infarction and CAD. The polymorphism’s correlation with high risk of the above diseases is particularly evident in the Korean and Chinese populations but not in the Japanese population.	The synergism of smoking and ALDH2 gene mutation has a greater impact on CAD risk than either factor alone by increasing the amount of reactive aldehydes.	Daily supplementation of essential AD2 by ALDH2-deficient individuals resulted in lower blood levels of acetaldehyde.	The presence of a dominant A allele in the genotype of individuals with the mutation is associated with a higher risk of CAD. Patients with a mutation in the ALDH2 gene consume significantly less alcohol than patients without the A allele.	The G487A of ALDH2 mutation significantly increases the risk of CAD.

ALDH2, aldehyde dehydrogenase 2; CAD, coronary artery disease.

**Table 3:** Contraindications to the provocation test [1].

Some of the Contraindications to Performing an Intracoronary Provocation Test
Pregnancy
Severe hypertension
Significant left coronary artery trunk stenosis
Advanced heart failure
Severe aortic stenosis

**Table 4:** Pharmacological treatment of CAS [1,4,5,26,83].

Medication	Mechanism	Effect
CCBs	Inhibition of L-type calcium channels of myocytes in vessels	Reduction of vascular resistance and relaxation of coronary arteries
nitrates	NO production as a result of transformations occurring in the vessel wall	Vasodilatation
statins	Inhibition of RhoA/ROCK pathway, increase in NO activity, decrease in ROS	CAS inhibition
aspirin	Blockage of thromboxane A2 production (dose < 100 mg per day)	Vasodilatation
Rho-kinase inhibitors	Inhibition of RhoA/ROCK pathway	Decreased contraction of VSMCs
nicorandil	Activation of nitrates and potassium channel	Dilation of the coronary arteries

CCBs, calcium channel blockers.

## Data Availability

The data used in this article are sourced from materials mentioned in the References section.
